# Mendelian randomization study on the causal link between reproductive behavior and postpartum depression

**DOI:** 10.1097/MD.0000000000044399

**Published:** 2025-09-12

**Authors:** Jinghui Zou, Cheng Li, Hong Ma, Yisheng Zhang

**Affiliations:** a Department of Obstetrics, The Affiliated Lihuili Hospital of Ningbo University, Ningbo, China; b Department of Thyroid and Breast Surgery, The Affiliated Lihuili Hospital of Ningbo University, Ningbo, China; c Department of Psychosomatic Medicine, The Affiliated Kangning Hospital of Ningbo University, Ningbo, China.

**Keywords:** age, first birth, first intercourse, Mendelian randomization, postpartum depression, sexual partners

## Abstract

This study employed Mendelian randomization (MR) to explore the causal relationships between age at first sexual intercourse (AFS), lifetime number of sexual partners (LNSP), age at first birth (AFB) and risk of postpartum depression (PPD). We used aggregated data from genome-wide association studies to analyze AFS, LNSP, and AFB as exposure variables, with PPD as the outcome variable. Single nucleotide polymorphisms (SNPs) strongly correlated with exposure variables were selected as instrumental variables. MR analysis was conducted using 5 methods: inverse-variance weighted (IVW), MR Egger, weighted median, simple, and weighted modes. The Cochran’s *Q* test was used to evaluate heterogeneity among SNPs. The MR-Egger intercept method assessed horizontal pleiotropy, whereas leave-one-out analysis evaluated the sensitivity of the causal association. IVW analysis revealed a significant negative causal relationship between AFS and PPD (OR = 0.417, 95% CI: 0.327–0.531, *P* < .001) and between AFB and PPD (OR = 0.842, 95% CI: 0.762–0.931, *P* < .001). However, there was a positive causal link between LNSP and PPD (OR = 1.965, 95% CI: 1.202–3.212, *P* = .007). The Cochran’s *Q* test for the causal link suggested heterogeneity among SNPs between AFS and PPD (*P* < .05) and LNSP and PPD (*P* < .05), focusing on the results of IVW. There was no heterogeneity in SNPs between the AFB and PPD groups (*P* *>* .05). The results of the MR-Egger intercept test showed no horizontal pleiotropy, and the leave-one-out analysis confirmed that the 3 causal links were robust. Our study demonstrated that AFS, LNSP, and AFB were causally associated with PPD risk. Early AFB, AFS, and increased LNSP are risk factors for PPD.

## 1. Introduction

Postpartum depression (PPD) refers to a series of mental symptoms such as low mood, diminished interest, slow thinking after childbirth, and suicidal tendencies in severe cases.^[1]^ PPD has become a global public health problem with an incidence of approximately 10% to 15%, but it is as high as 50% to 80% in low- and middle-income countries.^[2]^ PPD affects the health of mothers and children, not only damaging the mother herself but also affecting the emotional, behavioral, and cognitive development of the baby, increasing the risk of depression, autism, and attention deficit hyperactivity disorder in childhood.^[3,4]^ The pathogenesis of PPD is complex and involves multiple risk factors such as physiological, psychological, and social environments,^[5]^ history of depression or mental illness, excessive mental stress during pregnancy, stressful life events, lack of spousal support, and low socioeconomic status.^[6]^ Therefore, early identification of high-risk factors for PPD, targeted management, and intervention are crucial to the prevention of PPD.

Human reproductive behavior encompasses factors such as age at first sexual intercourse (AFS), lifetime number of sexual partners (LNSP), and age at first birth (AFB), etc.^[7]^ Epidemiological studies have shown that AFS tends to advance.^[8]^ A survey of 3848 students aged 15 to 24 found that 83% had sexual intercourse, and the average age was 16 years.^[9]^ Similarly, in the United Kingdom, 3 national behavioral and lifestyle surveys showed a marked increase in the proportion of teenagers who had sexual intercourse for the first time before the age of 16, as well as in the number of heterosexual partners.^[7]^ A cohort study from Brazil showed that the smaller the AFS, the more likely adolescents were to develop severe depression.^[10]^ Rubin et al^[11]^ identified a potential link between LNSP and depression in a predominantly African American cohort, although the evidence was deemed insufficient. These studies included participants from Europe, North America, and other parts of the world, with a degree of ethnic heterogeneity and inconsistent study methods, making it difficult to comprehensively evaluate the causal association between reproductive behavior and PPD.

Mendelian randomization (MR) studies utilize the principles of Mendelian genetics, specifically random assignment, and leverage genome-wide association studies (GWAS) data. By employing SNPs as instrumental variables, MR studies address confounding factors and biases inherent in traditional observational studies, thereby mitigating reverse causality.^[12]^ MR studies provide a more precise identification of potential causal links between exposure factors and outcome variables than randomized controlled trials.^[13]^ This study aimed to examine the causal relationships between AFS, LNSP, AFB, and PPD using MR, highlighting the unique value and necessity of MR analysis in addressing complex health issues and offering meaningful recommendations for the clinical prevention of PPD.

## 2. Materials and methods

### 2.1. Study design and data sources

**Figure 1.**
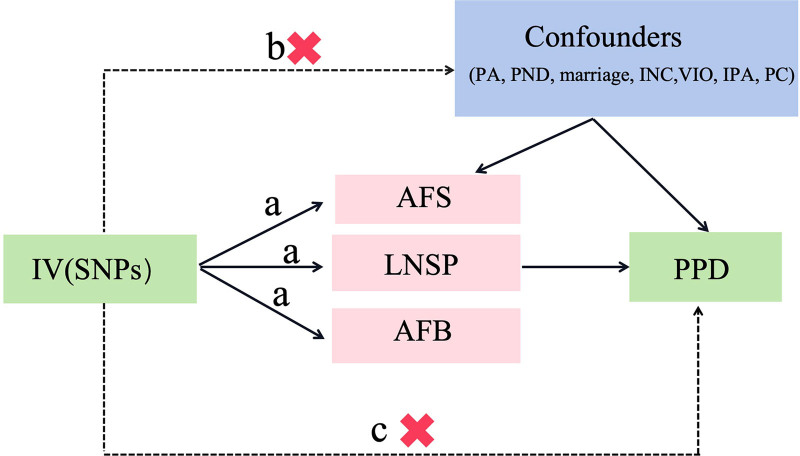
Three key assumptions of Mendelian randomization study.

This study implemented a 2-sample MR to examine the causative links among AFS, LNSP, AFB exposure and PPD. AFS, LNSP, and AFB served as exposure variables, with PPD as the outcome of interest. SNPs served as instrumental variables (IVs) for the analysis and were selected based on 3 key 2-sample MR criteria: a strong association between all selected IVs and AFS, LNSP, and AFB exposure (*P* < 5 × 10^−8^); independence of all selected IVs from confounders affecting AFS, LNSP, AFB, and PPD; and the influence of all selected IVs on PPD occurring exclusively through AFS, LNSP, and AFB, with no alternative mechanisms (Fig. 1).

GWAS data for AFS were obtained from the UK Biobank, contained 3,97,338 individuals (1,82,791 men and 2,14,547 women), including 1,63,59,424 SNPs.^[14]^ GWAS data for LNSP were obtained from UK Biobank’s 2018 large cohort study covering 3,78,882 participants of European descent, including 98,51,867 SNPs (https://gwas.mrcieu.ac.uk/datasets/ukb-b-4256/). GWAS data for AFB, sourced from the UK Biobank, included 5,42,901 European participants (1,24,088 men and 4,18,758 women) and encompassed 97,02,772 SNPs.^[14]^ GWAS data for PPD were sourced from the Finnish Biobank, comprising 67,205 participants of European ancestry, including 7604 PPD patients and 59,601 controls, with 1,63,76,275 SNPs (https://gwas.mrcieu.ac.uk/datasets/finn-b-O15 POSTPART DEPR/).

### 2.2. Selecting instrument variables

The MR analysis required strict adherence to 3 fundamental principles: relevance, independence, and exclusion. Consequently, each selected IV was subjected to thorough evaluation. SNPs significantly associated with AFS, LNSP, and AFB exposure (*P* < 5 × 10^−8^) were chosen, excluding those with *F*-values below 10, to ensure significance and minimize weak IV bias. The *F*-statistic was calculated using the formula *F* = *R*^2^ × (N – 2)/(1 – *R*^2^), where *R*^2^ denotes the proportion of variance in AFB explained by each independent variable. *R*^2^ is determined by multiplying the square of Beta, which indicates the allelic impact, by twice the product of the effect allele frequency and its complement (1 − EAF). A clumping procedure (*r*^2^ < 0.001, physical distance = 10,000 kb) was employed to eliminate biases from strong linkage disequilibrium among SNPs, ensuring the independence of instrumental variables. Palindromic SNPs with intermediate allele frequencies were excluded to ensure consistent alignment of effect alleles across the AFS, LNSP, AFB, and PPD datasets.

### 2.3. Statistical analysis

The genetic connection among AFS, LNSP, AFB, and PPD were examined using MR-Egger regression, weighted median, inverse-variance weighted (IVW), simple mode, and weighted mode methods. IVW has been established as the principal method because it has unbias and the highest statistical power when the instrumental variables are valid, and reduced the influence of measurement errors or sample heterogeneity on the overall estimation under the assumption of pleiotropy balance. MR-Egger and the weighted median were used as supplements to the sensitivity analysis to evaluate the reliability of the IVW results.^[15]^ The Cochran’s *Q* test assessed SNPs heterogeneity, with *P* < .05 indicating statistical heterogeneity. The leave-one-out method was used for sensitivity analysis to assess how removing different SNPs affected the causal effect, ensuring MR stability. The MR-Egger intercept and MR-PRESSO tests assessed SNP polymorphism and outliers. The MR-Egger intercept was significantly different from 0 (*P* < .05), indicating that the results had horizontal pleiotropy. Data analysis were conducted using the TwoSampleMR, MR-PRESSO, and MR package in R software (v4.3.2).

## 3. Results

### 3.1. Choosing instrumental variable

AFS, LNSP, AFB all showed the significance of whole-genome studies (*P* < 5 × 10^−8^).^[16]^ The linkage imbalance coefficient (*r*^2^ < 0.001) was used to ensure that the SNPs were independent of each other and to minimize gene pleiotropy the impact of the results, with a physical window of 10,000 kb.^[17]^ SNPs highly correlated with exposure factors were screened in the PPD dataset, SNPs with high linkage were replaced with missing SNPs, and SNPs without substitution sites were excluded. The instrumental variable impact of each SNP was assessed using the *F* statistic, calculated as *F* = *R*^2^ (N – 2)/(1 – *R*^2^), which does not have a weak variable effect when *F* > 10.^[18]^

For subsequent MR analysis, 196 SNPs related to AFS were included, after AFS data matched with PPD data, 31 palindromic SNPs with intermediate alleles and 1 SNPs with repeated names were deleted, and finally 143 SNPs related to AFS were included as instrumental variables. Using the same methodology, 57 SNPs related to LNSP and 50 SNPs related to AFB were included as instrumental variables.

### 3.2. Mendelian randomization analysis

**Figure 2.**
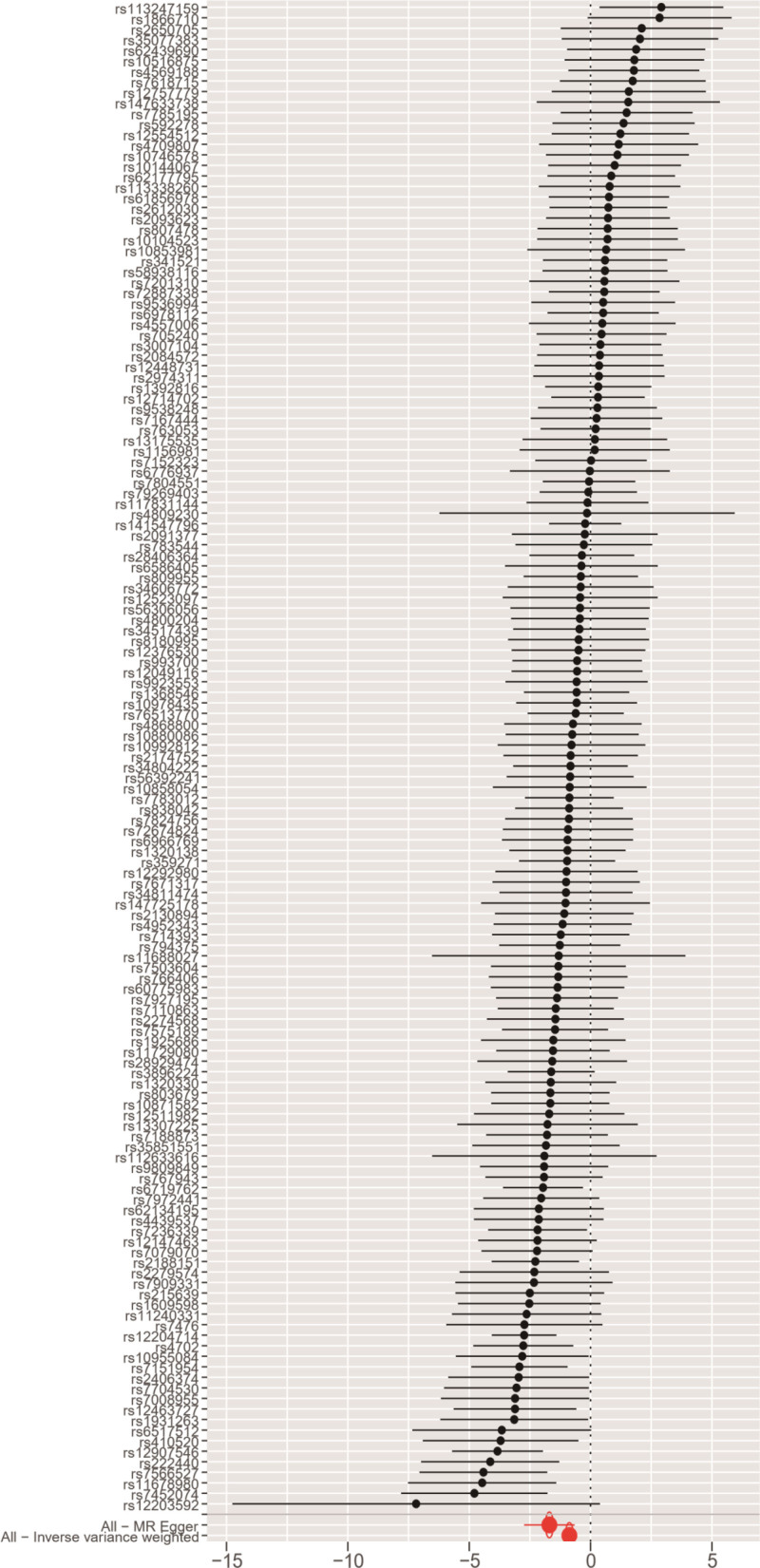
Forest plot of the effect of AFS on PPD. *Y*-axis represented the analyzed SNPs; black dots represented the causal effect estimate of PPD risk with the increase of AFS; two red dots represented 2 different methods; the horizontal black lines represented the 95% confidence intervals of the causal estimate. AFS = age at first sexual intercourse, MR = Mendelian randomization, PPD = postpartum depression, SNPs = single nucleotide polymorphisms.

The MR method was used to examine the causal link between AFS and PPD. Both the IVW and Weighted median methods indicated a negative causal association between AFS and PPD with the IVW method, which was statistically significant (*P* < .001, 95% CI = 0.417[0.327–0.531]), suggesting that a decrease in AFS is linked to a higher risk of PPD (Table 1 and Fig. 2).

**Table 1:** The MR results between AFS and PPD by 5 methods.

Exposure	Outcome	Method	β	SNP (n)	OR	95% CI	*P*-value
AFS	PPD	MR-Egger	−1.699	143	0.183	0.064, 0.519	.002
AFS	PPD	Weighted median	−0.824	143	0.438	0.314, 0.612	<.001
AFS	PPD	IVW	−0.875	143	0.417	0.327, 0.531	<.001
AFS	PPD	Simple mode	−0.765	143	0.465	0.183, 1.183	.110
AFS	PPD	Weighted mode	−0.661	143	0.516	0.188, 1.422	.203

AFS = age at first intercourse, CI = confidence interval, IVW = inverse-variance weighted, MR = Mendelian randomization, OR = odds ratio, PPD = postpartum depression, SNP (n) = number of single-nucleotide polymorphism.

There was heterogeneity among SNPs (Cochran’s *Q* test, *Q* = 174.477.

**Figure 3.**
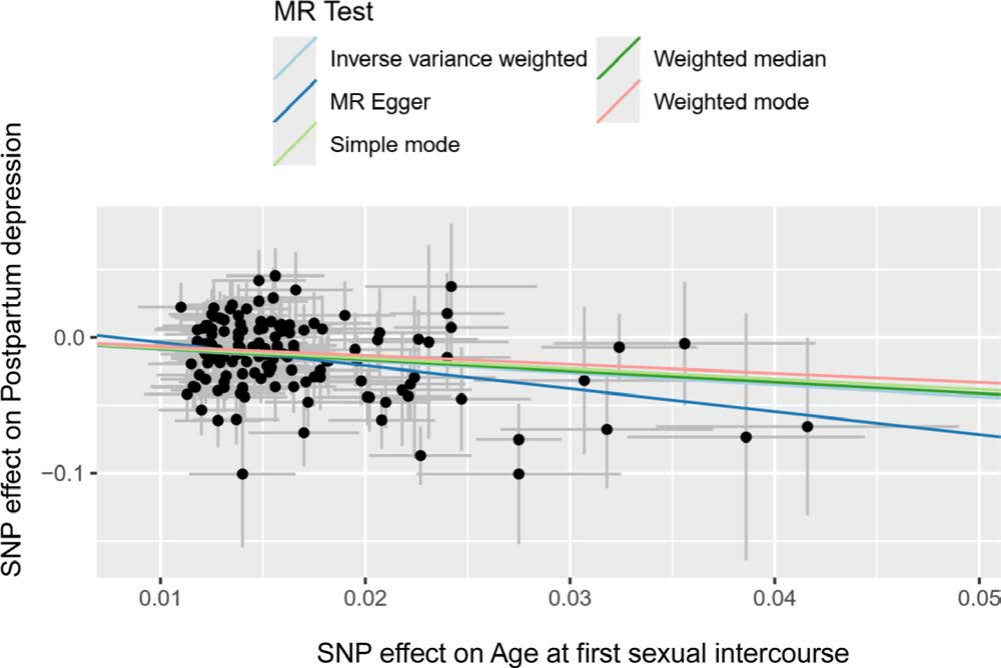
The scatter plot shows the causal effect of AFS on PPD. *X*-axis represented instrumental variables related to AFS; *Y*-axis represented instrumental variables related to PPD; black dots represent the causal effect estimate of PPD risk with the increase of AFS. AFS = age at first sexual intercourse, MR = Mendelian randomization, PPD = postpartum depression.

The analysis (*P* = .029) suggested varying effects of different SNPs on the outcome. However, the MR Steiger test confirmed the consistency of these variables with the causal directionality between AFS and PPD (*P* < .05), supporting the reliability of the IVW results. In addition, the scatter plot results showed that the SNPs closely related to AFS and PPD were stable (Fig. 3).

**Figure 4.**
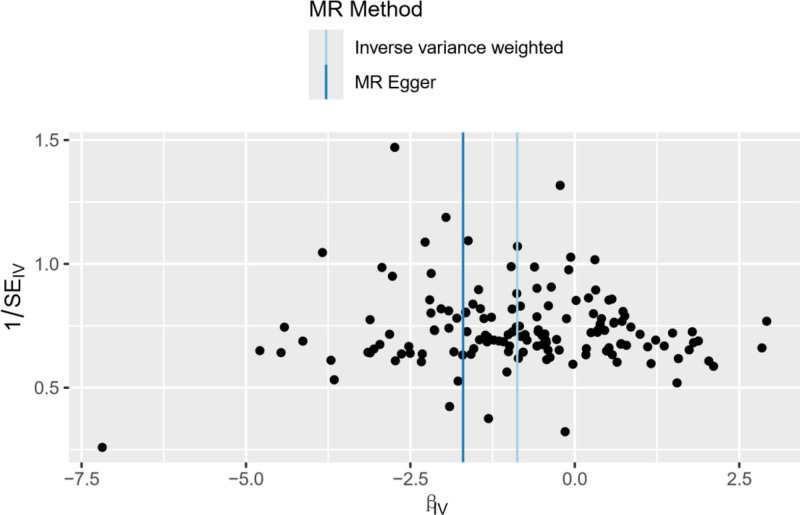
Funnel plot of the effect of AFS on PPD. βIV, the effect size of the allele; 1/SEIV, the standard error of the effect size of the allele. AFS = age at first sexual intercourse, MR = Mendelian randomization, PPD = postpartum depression.

**Figure 5.**
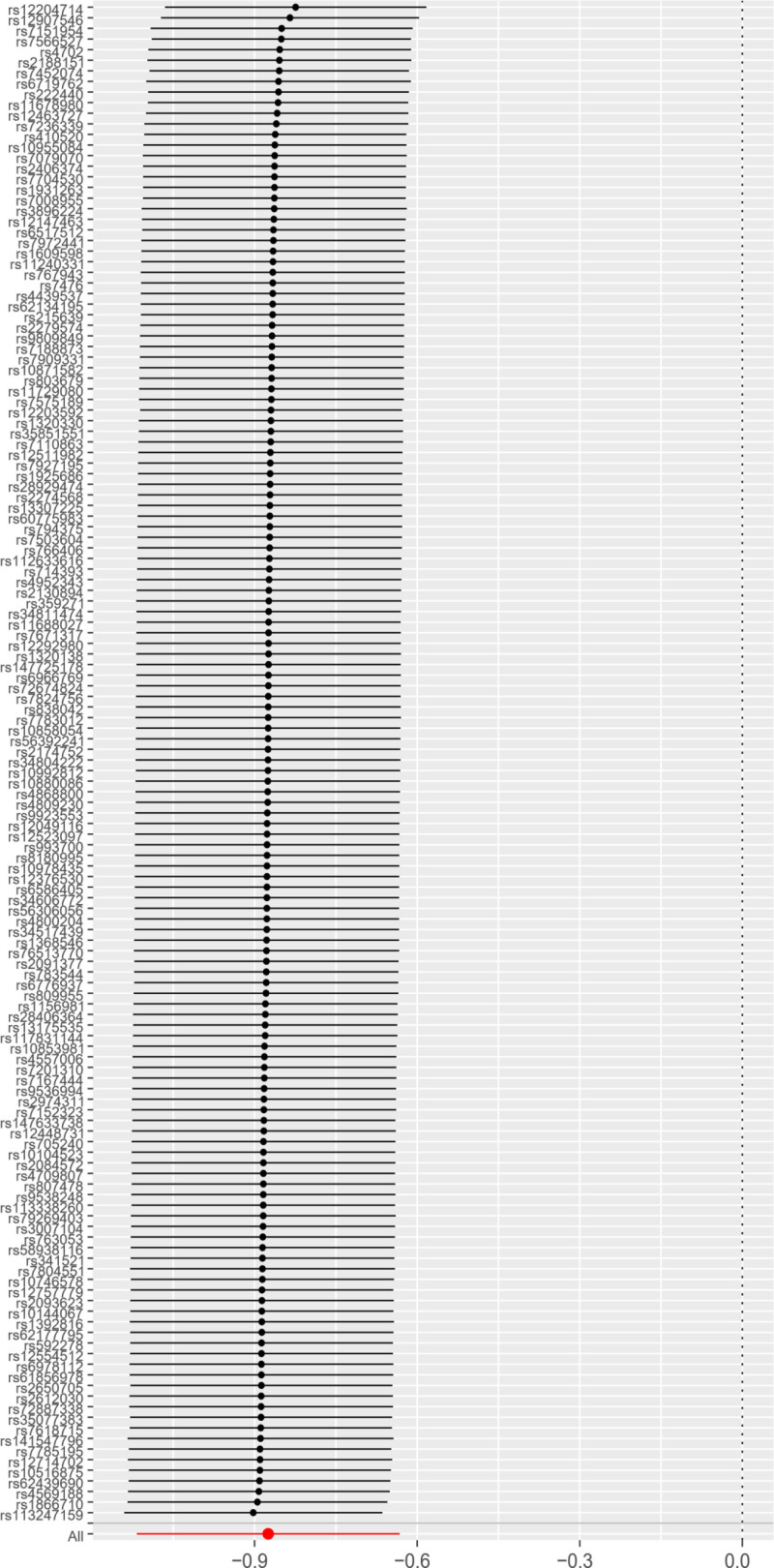
Leave-one-out shows the sensitivity analysis of AFS for PPD. The black dots represented the causal effect estimate when excluding the SNP and using the remaining SNPs as instrumental variables; the horizontal black lines represented the 95% confidence intervals of the causal estimate. AFS = age at first sexual intercourse, MR = Mendelian randomization, PPD = postpartum depression, SNPs = single nucleotide polymorphisms.

The evaluation of pleiotropy using funnel plots indicated symmetry in the SNPs, suggesting no pleiotropy in the MR analysis (Fig. 4). Additionally, the MR-Egger intercept and MR-PRESSO analyses revealed no significant pleiotropy or outliers (*P* = .114), reinforcing the reliability of the causal inference. The leave-one-out analysis demonstrated that removing each SNP sequentially resulted in all error lines remaining on the left side of 0, suggesting no SNPs significantly influenced the evaluation outcomes (Fig. 5). This indicates a robust causal association between AFS and PPD.

**Figure 6.**
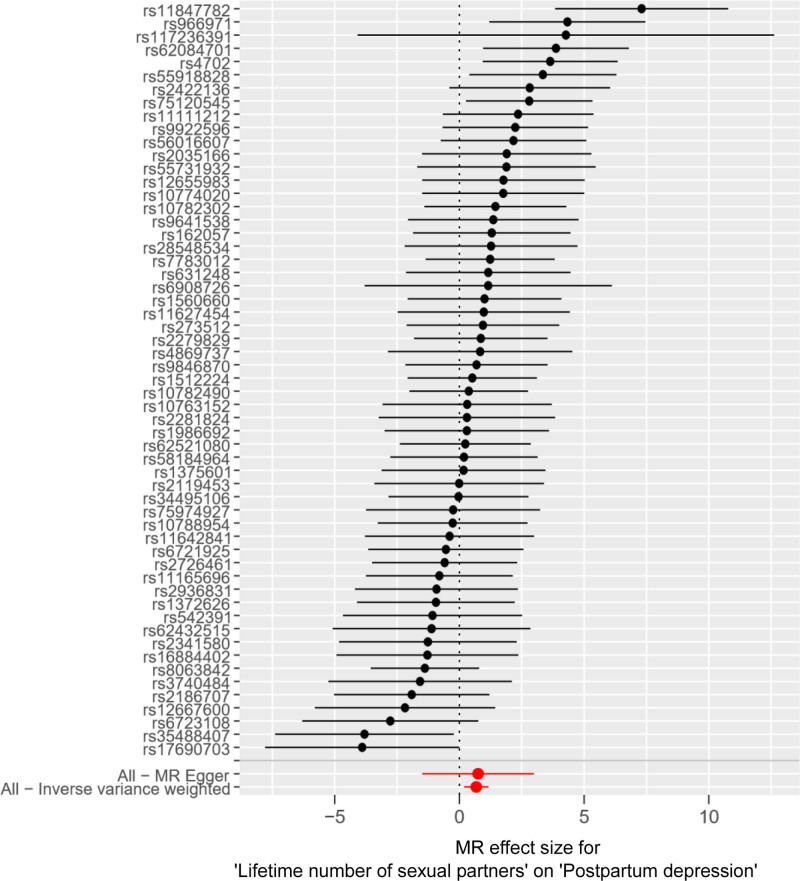
Forest plot of the effect of LNSP on PPD. *Y*-axis represented the analyzed SNPs; black dots represented the causal effect estimate of PPD risk with the increase of LNSP; two red dots represented 2 different methods; the horizontal black lines represented the 95% confidence intervals of the causal estimate. AFS = age at first sexual intercourse, MR = Mendelian randomization, PPD = postpartum depression, SNPs = single nucleotide polymorphisms.

IVW results showed a positive causal association between LNSP and PPD (*P* = .007, 95% CI = 1.965[1.202–3.212]; Table 2 and Fig. 6).

**Table 2:** The MR results between LNSP and PPD by 5 methods.

Exposure	Outcome	Method	β	SNP (n)	OR	95% CI	*P*-value
LNSP	PPD	MR-Egger	0.750	57	2.116	0.225, 19.897	.515
LNSP	PPD	Weighted median	0.478	57	1.613	0.872, 2.985	.128
LNSP	PPD	IVW	0.676	57	1.965	1.202, 3.212	.007
LNSP	PPD	Simple mode	0.649	57	1.913	0.465, 7.881	.373
LNSP	PPD	Weighted mode	0.557	57	1.746	0.456, 0.682	.419

CI = confidence interval, IVW = inverse-variance weighted, LNSP = lifetime number of sexual partners, MR = Mendelian randomization, OR = odds ratio, PPD = postpartum depression, SNP (n) = number of single-nucleotide polymorphism.

**Figure 7.**
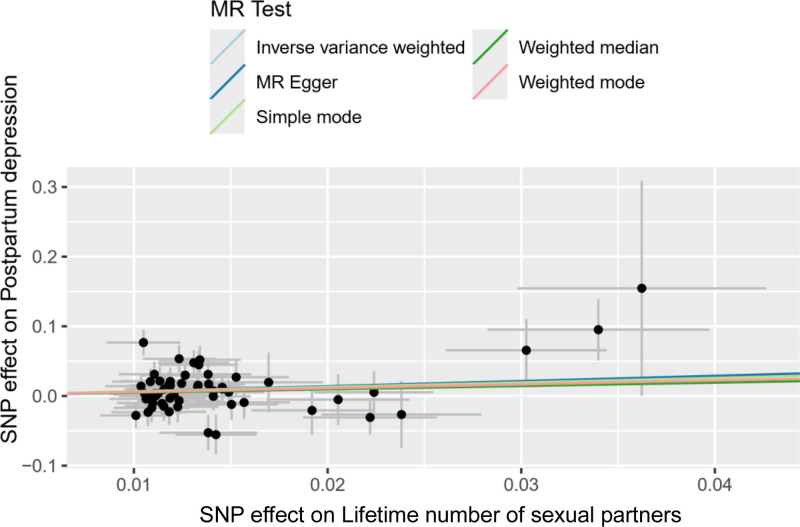
The scatter plot shows the causal effect of LNSP on PPD. *X*-axis represented instrumental variables related to LNSP; *Y*-axis represented instrumental variables related to PPD; black dots represent the causal effect estimate of PPD risk with the increase of LNSP. LNSP = lifetime number of sexual partners, MR = Mendelian randomization, PPD = postpartum depression.

To detect heterogeneity, the Cochran’s *Q* test was conducted. The MR-Egger approach produced a *Q* statistic of 77.907 (*P* = .023) indicating heterogeneity among SNPs. The steiger test showed that these variables were consistent with the directionality of the causal association between LNSP and PPD (*P* < .05), indicating that the IVW results were still reliable, and the scatter plot results showed that SNPs closely related to LNSP and PPD were stable (Fig. 7).

**Figure 8.**
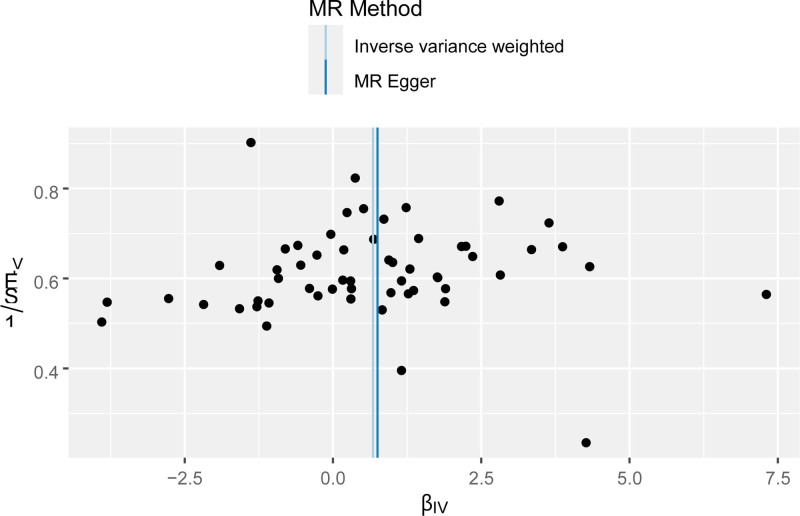
Funnel plot of the effect of LNSP on PPD. βIV, the effect size of the allele; 1/SEIV, the standard error of the effect size of the allele. LNSP = lifetime number of sexual partners, MR = Mendelian randomization, PPD = postpartum depression.

**Figure 9.**
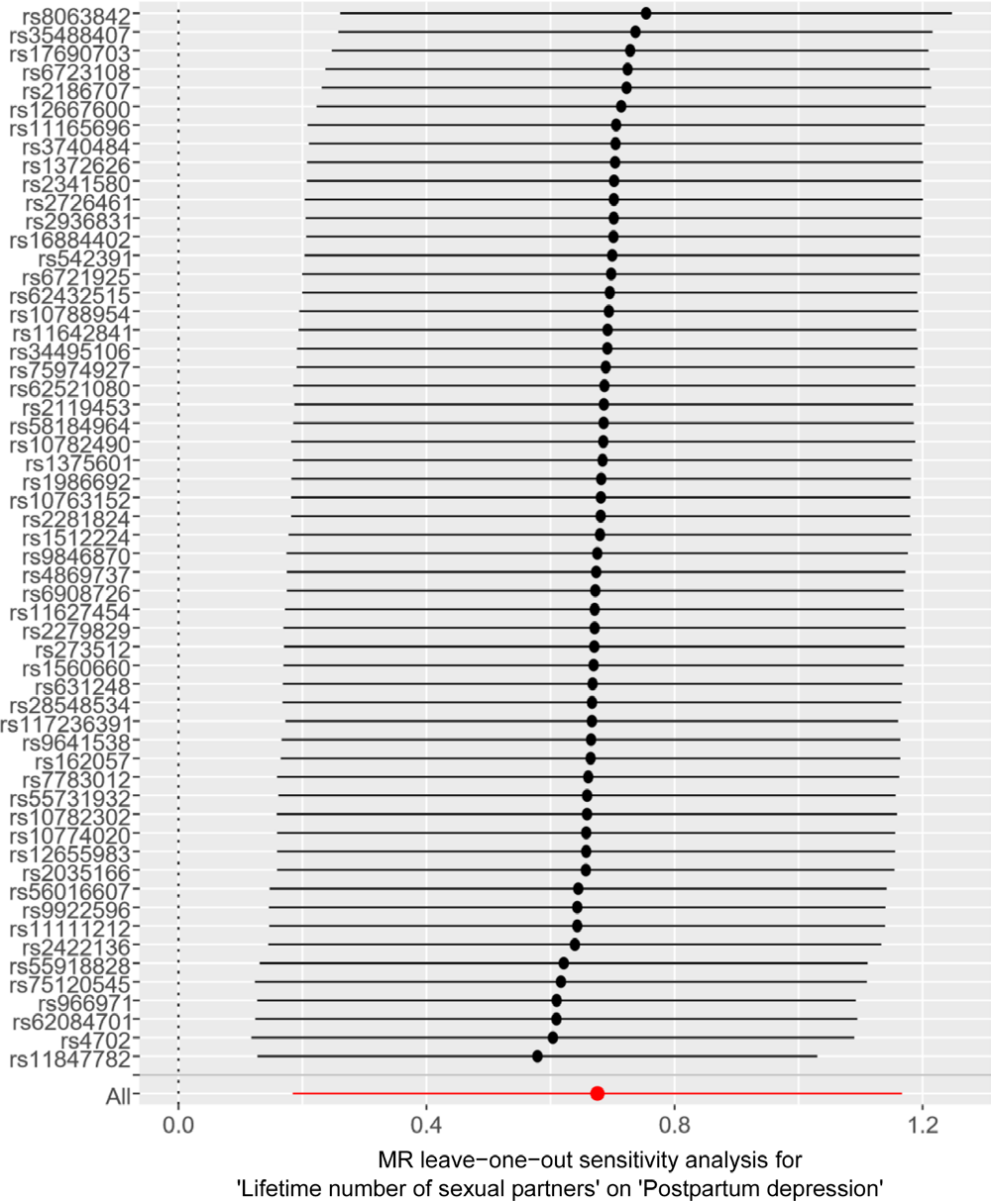
Leave-one-out shows the sensitivity analysis of LNSP for PPD. The black dots represented the causal effect estimate when excluding the SNP and using the remaining SNPs as instrumental variables; the horizontal black lines represented the 95% confidence intervals of the causal estimate. LNSP = lifetime number of sexual partners, MR = Mendelian randomization, PPD = postpartum depression, SNPs = single nucleotide polymorphisms.

The funnel plot indicated symmetry for the SNP (Fig. 8), and both MR-Egger intercept and MR-PRESSO analysis revealed no significant pleiotropy or outliers (*P* = .634), confirming the absence of pleiotropy in the MR analysis. Leave-one-out analysis revealed that no single SNP significantly influenced the evaluation outcomes (Fig. 9), suggesting a stable and reliable causal link between LNSP and PPD.

**Figure 10.**
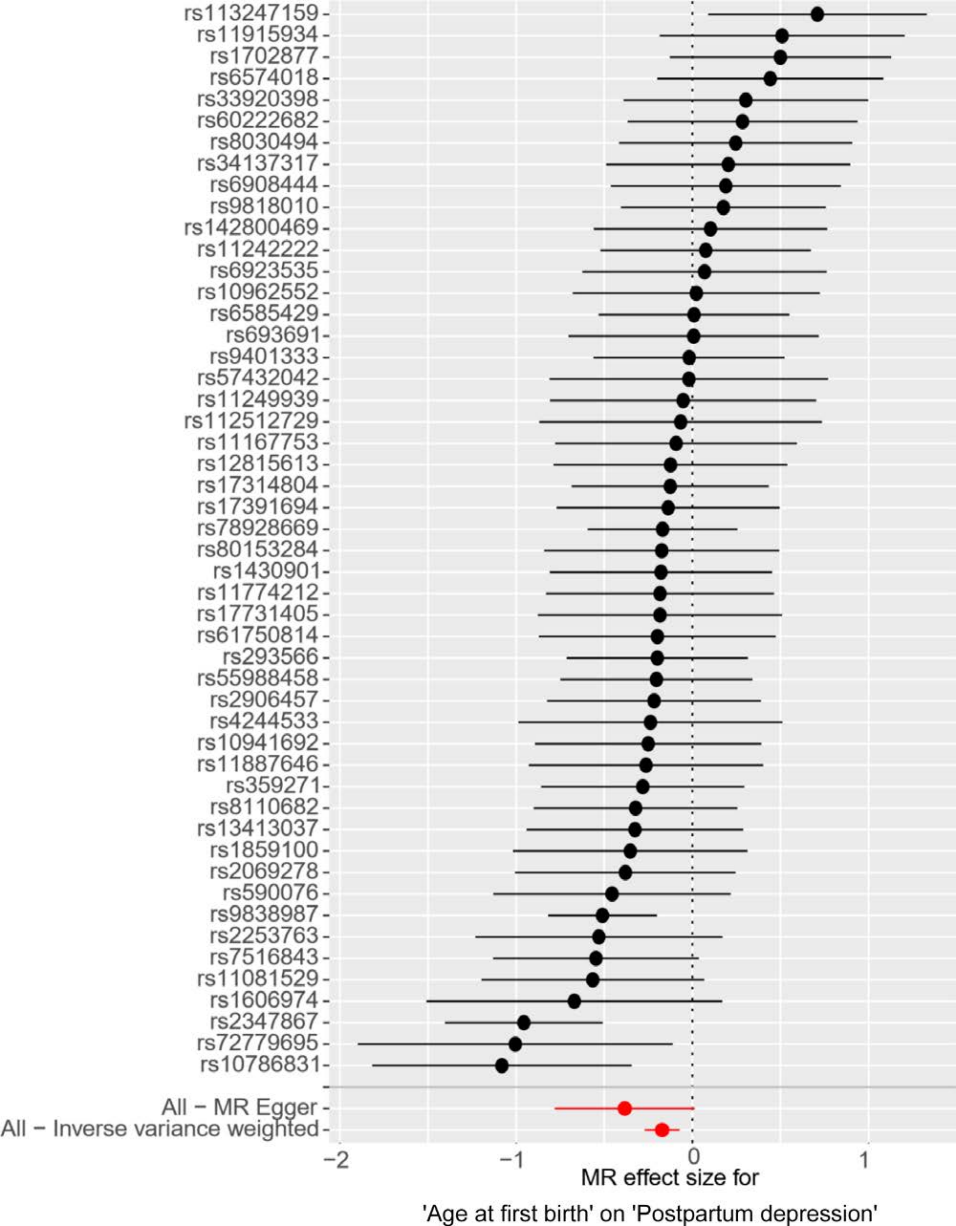
Forest plot of the effect of AFB on PPD. *Y*-axis represented the analyzed SNPs; black dots represented the causal effect estimate of PPD risk with the increase of AFB; two red dots represented 2 different methods; the horizontal black lines represented the 95% confidence intervals of the causal estimate. AFB = age at first birth, IVW = inverse variance weighted, MR = Mendelian randomization, PPD = postpartum depression.

IVW results showed a positive causal association between AFB and PPD (*P* < .001, 95% CI = 0.842[0.762–0.931]; Table 3 and Fig. 10).

**Table 3:** The MR results between AFB and PPD by 5 methods.

Exposure	Outcome	Method	β	SNP (n)	OR	95% CI	*P*-value
AFB	PPD	MR-Egger	−0.384	50	0.681	0.485, 1.013	.064
AFB	PPD	Weighted median	−0.177	50	0.838	0.735, 0.955	.008
AFB	PPD	IVW	−0.171	50	0.842	0.762, 0.931	<.001
AFB	PPD	Simple mode	−0.169	50	0.845	0.622, 1.147	.284
AFB	PPD	Weighted mode	−0.178	50	0.837	0.631, 1.110	.221

AFB = age at first birth, CI = confidence interval, IVW = the inverse-variance weighted method, MR = Mendelian randomization, OR = odds ratio, PPD = postpartum depression, SNP (n) = number of single-nucleotide polymorphism.

**Figure 11.**
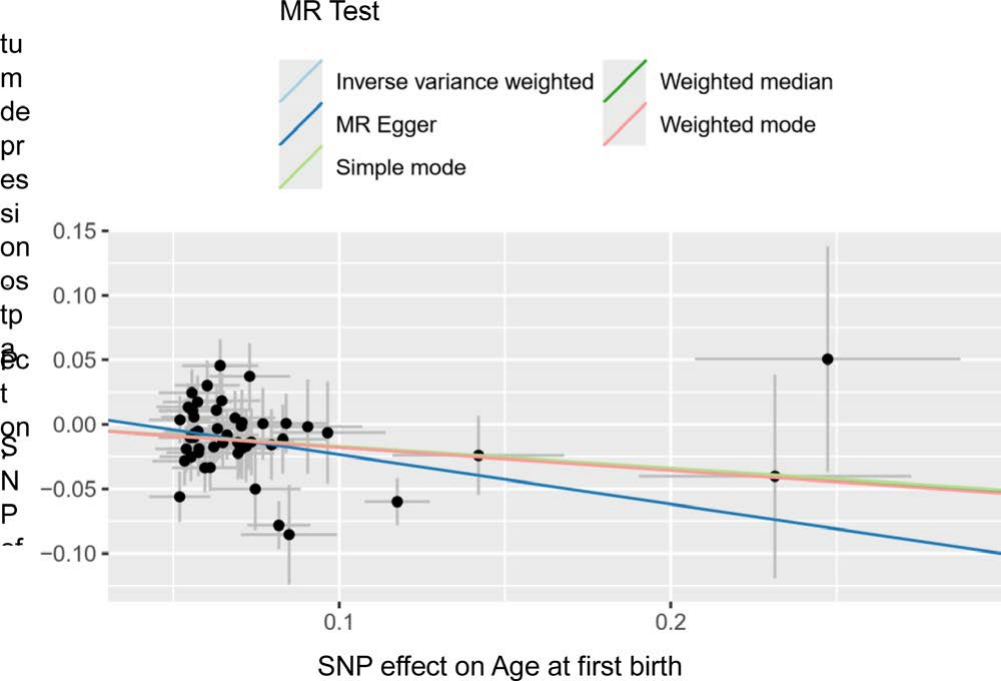
The scatter plot shows the causal effect of AFB on PPD. *X*-axis represented instrumental variables related to AFB; *Y*-axis represented instrumental variables related to PPD; black dots represent the causal effect estimate of PPD risk with the increase of AFB. AFB = age at first birth, IVW = inverse variance weighted, MR = Mendelian randomization, PPD = postpartum depression.

**Figure 12.**
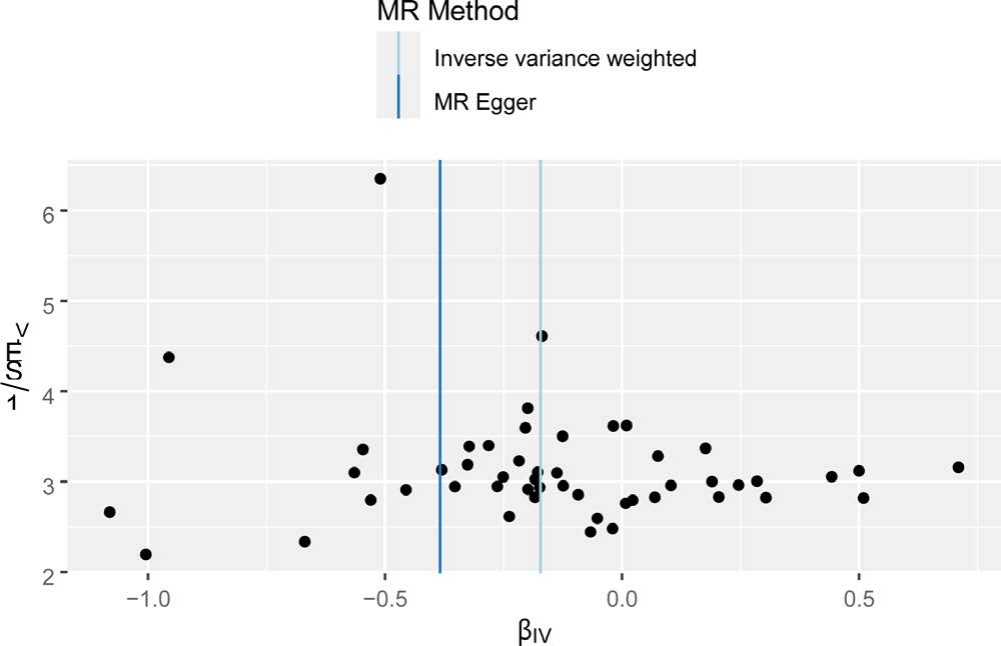
Funnel plot of the effect of AFB on PPD. βIV, the effect size of the allele; 1/SEIV, the standard error of the effect size of the allele. AFB = age at first birth, IVW = inverse variance weighted, MR = Mendelian randomization, PPD = postpartum depression.

**Figure 13.**
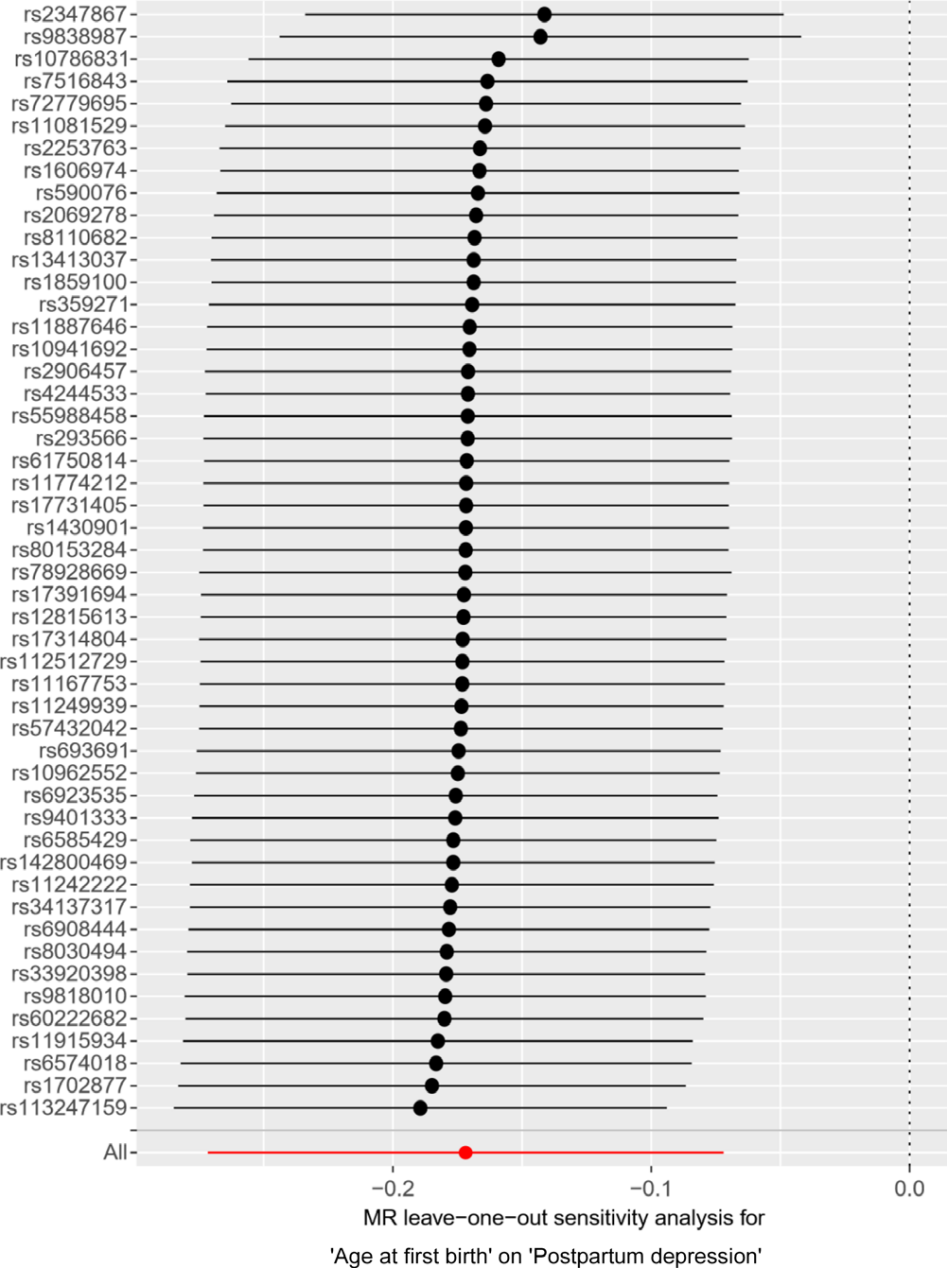
Leave-one-out shows the sensitivity analysis of AFB for PPD. The black dots represented the causal effect estimate when excluding the SNP and using the remaining SNPs as instrumental variables; the horizontal black lines represented the 95% confidence intervals of the causal estimate. AFB = age at first birth, MR = Mendelian randomization, PPD = postpartum depression, SNPs = single nucleotide polymorphisms.

Sensitivity analysis of the MR analysis showed no heterogeneity among SNPs (Cochran’s *Q* test, *Q* = 63.417, *P* = .067). The scatter plot (Fig. 11) indicates the stability of SNPs associated with AFB and PPD. The symmetric funnel plot (Fig. 12), along the MR-Egger intercept and MR-PRESSO analysis (*P* = .285), suggests no significant pleiotropy or outliers, confirming the absence of pleiotropy in the MR analysis. The leave-one-out method indicated that the causal link between AFB and PPD was relatively stable and reliable (Fig. 13).

## 4. Discussion

This study revealed that early AFS, AFB, and increased LNSP are risk factors for PPD by MR methods. Moreover, both the MR-Egger intercept method and leave-one methods confirm that the MR results of this study are robust. This means that delaying sexual intercourse and childbearing age appropriately, reducing the number of sexual partners, and promoting healthy sexual behavior may help reduce the incidence of PPD. This conclusion is of great significance to further study the biological mechanism between reproductive behavior and PPD, and the prevention of PPD.

Previous observational studies have shown that individuals with AFS who are 12 to 14 years old and have had 2 or more sexual partners have a higher risk of depression.^[19]^ McMahon et al^[20]^ have suggested a negative correlation between AFB and perinatal depression. A Swedish cohort of 11,253 twins showed an association between early sexual activity (<16 years of age) and subsequent social maladjustment and depression.^[21]^ The finding is similar to the results of the present study. However, Aasheim V et al^[22]^ found that women of AFB over 32 years had a higher risk of perinatal depression than those aged 25 to 31 years. A study in Turkey showed no association between AFB and PPD.^[23]^ Epidemiological studies on the causal relationship between reproductive behavior and PPD have been hampered by family confounding and other factors,^[21]^ so it is always controversial. Recent MR studies indicate that early AFS and AFB are risk factors for major depressive disorder (MDD).^[24]^ PPD is frequently characterized as a postpartum episode of MDD^[25]^ and can manifest as complex phenotypes of various diseases via distinct pathways.^[26,27]^ Lu Zhe et al^[28]^ have also confirmed a negative causal relationship between AFS and MDD through MR analysis (*P* < .001, 95% CI = 0.720[0.661–0.784]), but a positive causal association between LNSP and MDD (*P* < .001, 95% CI = 1.656[1.356–2.022]). These findings are consistent with our findings.

The exact pathological mechanism between AFS, AFB, LNSP, and PPD was presently unclear.^[29]^ Adolescence is a time of increased risk-taking behavior, such as risky sexual behavior (including not using condoms and high-risk partners, etc), smoking, drug and alcohol abuse, and intentional self-harm.^[30]^ Because the adolescent brain, the higher functional areas related to impulsive behavior control, are not mature,^[31]^ AFS is too early, and it is more prone to increased LNSP number, infrequent condom use, unintended pregnancy, early AFB, and sexually transmitted diseases.^[32]^ These pregnant adolescents will face significant changes in their educational, family and professional roles, which exerts considerable pressure on their mental health. Mental stress may also increase the risk of PPD.^[33]^ Lack of economic support, marital pressure and domestic violence faced by may also exacerbate PPD in young mothers.^[34]^ The brains of adolescents are undergoing a sensitive developmental stage of synaptic pruning.^[35]^ Environmental stress and elevated cortisol levels can disrupt the synaptic structure and function of the hippocampus, prefrontal cortex and amygdala, which may lead to PPD.^[35]^ In addition, childbirth is regarded as the main trigger of PPD. The decrease in allopregnanolone levels after delivery is closely related to the onset of PPD by affecting the activity of gamma-aminobutyric acid receptor.^[33]^ However, women of early AFB may experience a more significant reduction in allopregnanolone thereby increasing the risk of PPD.

There are some limitations to our study. First, the participants in this study were entirely of European descent, and whether the findings can be generalized to other populations and regions needs to be generalized. Horizontal pleiotropy cannot be completely ruled out, necessitating the confirmation of our MR results through high-quality prospective epidemiological studies. Our study did not include detailed data on age and number of sexual partners due to privacy restrictions in the UK Biobank. Consequently, we were unable to perform stratified analyses of AFS, AFB, and LNSP and or to assess the influence of exposure factors on PPD outcomes through subgroup analysis. Furthermore, individual genetic variations do not adequately capture the complexity of the specific biological indicators. Future studies should also explore in depth the possible mediating variables for the causal association between reproductive behavior and PPD. With the continuous expansion of the scope of global GWAS research scope, future studies are expected to solve this problem.

This study is the first to demonstrate a significant causal link between 3 reproductive behaviors and PPD risk using MR analysis, offering robust genetic evidence for PPD prevention. Therefore, we emphasize the importance of early sex education for adolescent girls, screening and monitoring for depression in sexually active adolescent girls at an early age, and providing psychoeducational interventions on depressive symptoms and sexual risk to improve sexual behavior and reproductive health, thereby reducing the incidence of PPD.

## Author contributions

**Conceptualization:** Jinghui Zou, Yisheng Zhang.

**Data curation:** Jinghui Zou.

**Methodology:** Jinghui Zou, Cheng Li, Hong Ma.

**Software:** Cheng Li.

**Supervision:** Hong Ma, Yisheng Zhang.

**Writing – review & editing:** Yisheng Zhang.
